# Abdominal obesity in Chinese patients undergoing hemodialysis and its association with all-cause mortality

**DOI:** 10.3389/fendo.2023.1287834

**Published:** 2023-10-26

**Authors:** Zhihua Shi, Yidan Guo, Pengpeng Ye, Yang Luo

**Affiliations:** ^1^ Division of Nephrology, Beijing Shijitan Hospital, Capital Medical University, Beijing, China; ^2^ Division of Injury Prevention and Mental Health, National Center for Chronic and Non-communicable Disease Control and Prevention, Chinese Center for Disease Control and Prevention, Beijing, China

**Keywords:** abdominal obesity cohort study, hemodialysis, waist circumference, body mass index, all-cause mortality

## Abstract

**Introduction:**

Obesity in patients undergoing hemodialysis is common. However, there is limited information on the relationship between obesity types defined by the combined body mass index (BMI) and waist circumference (WC) classification criteria and all-cause mortality in Chinese hemodialysis patients. Our objective was to determine the association between obesity types and all-cause mortality in hemodialysis patients.

**Methods:**

We conducted a prospective cohort study including patients from 11 hemodialysis centers in Beijing. According to the World Health Organization’s standards, patients were classified into 2 categories with WC and 4 categories with BMI and then followed up for 1 year. Kaplan–Meier survival analysis was used to compare the difference in the cumulative survival rate in different BMI and WC groups. A multivariate Cox regression analysis was used to determine the association between different types of obesity and all-cause mortality.

**Results:**

A total of 613 patients were enrolled, the mean age was 63.8 ± 7.1 years old, and 42.1% were women. Based on the baseline BMI, there were 303 (49.4%) patients with normal weight, 227 (37.0%) with overweight, 37(6.0%) with obesity, and 46 (7.5%) with underweight. Based on the baseline WC, 346 (56.4%) patients had abdominal obesity. During a median follow-up of 52 weeks, 69 deaths occurred. Kaplan–Meier plots demonstrated a significant association of BMI categories (log-rank χ2 = 18.574, *p*<0.001) and WC categories (log-rank χ2 = 5.698, *p*=0.017) with all-cause death. With normal BMI and non-abdominal obesity as a reference, multivariate Cox regression analysis results showed that obesity (HR 5.36, 95% CI, 2.09-13.76, *p*<0.001), underweight (HR, 5.29, 95% CI, 2.32-12.07, *p*<0.001), normal weight combined with abdominal obesity (HR 2.61, 95% CI, 1.20-5.66, *p*=0.016), and overweight combined with abdominal obesity (HR 1.79, 95% CI, 1.03-3.73, *p*=0.031, respectively) were significantly associated with higher risks of all-cause mortality.

**Conclusion:**

Our study indicated that abdominal obesity is common and associated with all-cause mortality among Chinese hemodialysis patients.

## Introduction

Obesity has reached epidemic proportions worldwide, and concerns about its influence on adverse outcomes have kept rising in the last decades (1, 2). In the general population, increased body mass index (BMI) has been shown to be an independent risk factor for all-cause mortality (3, 4). Compared with that in the general population, the results regarding this association in patients undergoing hemodialysis were different, a recent meta-analysis showed that BMI-defined obesity in hemodialysis patients was actually associated with reduced all-cause mortality, challenging the paradigm that obesity is related to increased mortality in the general population, and this inconsistent result was also called the “obesity paradox” (5). However, such a significantly different association in hemodialysis patients should not be simply explained as increased BMI is a protective factor, some studies in hemodialysis patients couldn’t validate this conclusion because of the existence of obese sarcopenia or protein-energy malnutrition (6).

Previous data showed that the use of BMI as a measurement of obesity has an important limitation because it does not discriminate abdominal from gluteofemoral fat (5, 7). To find more incremental risk information beyond the measurement of BMI alone among the obesity population, waist circumference (WC) measurement was introduced to assess abdominal obesity in various studies from different ethnic populations, and increased WC was also proved to be an independent risk factor of all-cause mortality even among normal-weight or overweight individuals in the general populations and peritoneal dialysis patients, however, there is still no information about the combination use of BMI and WC in the prediction of adverse outcomes among Chinese hemodialysis patients (8–11).

Compared with that in the general populations, the information about the characteristics of obesity and its influence on clinical adverse outcomes among elder hemodialysis patients is scarce, most previous studies about the relationship between obesity and mortality in hemodialysis patients were conducted with the measurement of BMI (12, 13),. There was still limited information about the association between abdominal obesity and all-cause mortality by combining measurements of BMI and WC in older hemodialysis patients in China. Therefore, we determined to investigate the characteristics of obesity and explore the association between abdominal obesity and all-cause mortality in a prospective cohort study of Chinese older patients undergoing hemodialysis.

## Materials and methods

### Ethics declaration and participants

The study protocol was approved by the Institutional Ethical Review Board of Beijing Shijitan Hospital, Capital Medical University (Approval No. SJT2016-18). We also performed the STROBE checklist for cohorts (supplementary STROBE checklist). All participants provided written informed consent for this study by themselves or their legal guardians. The patients’ identification numbers were anonymized to protect individuals’ privacy.

The data used in this study were obtained from a prospective cohort study: the cognitive impairment in Chinese hemodialysis patients (Registered in ClinicalTrials.gov, ID: NCT03251573). The cohort included 613 patients between the ages of 50 and 83 years from 11 hemodialysis centers in Beijing between April and June 2017, diagnosed with end-stage renal disease, treated with maintenance hemodialysis for a minimum of 3 months, and followed up for 1 year for the outcome of all-cause mortality (14). The inclusion criteria were as follows (1): age ≥50 years (2), end-stage renal disease with maintained hemodialysis treatment for a minimum of 3 months, and (3) willing to join the study and provide written informed consent. The exclusion criteria were as follows (1): estimation of a life expectancy of six months or less according to the nephrologists, and (2) planned kidney transplantation within 6 months of baseline.

Participants’ baseline characteristics were obtained from patient’s medical charts at the time of enrollment, including demographics (age, sex), lifestyle characteristics (smoking and alcohol intake), medical history (diabetes, hypertension, stroke, and coronary heart disease [CHD]) and laboratory variables (hemoglobin, albumin, total cholesterol, triglyceride, serum creatinine, blood urea nitroge, calcium, phosphate, intact parathyroid hormone [iPTH] and C reactive protein [CRP]). The single-pool Kt/V was calculated from the pre-and post-dialysis serum urea nitrogen levels as we applied in the previous study (15). Baseline CHD was defined as a history of recognized myocardial infarction, angina, and prior coronary angioplasty or bypass procedures.

### Definition of BMI and WC

In our study, we used BMI and WC measured at baseline as the indexes of obesity. All of the patients had received maintenance hemodialysis for at least 3 months to avoid the influence of fluid overload at the initial stage of the hemodialysis that would distort BMI and WC measurements. The anthropometric data (height, weight, and waist circumference) were measured after a dialysis session to avoid inaccurate that may be caused by fluid overload before dialysis. BMI was calculated as weight in kilograms divided by height in meters squared. Training personnel measured WC in centimeters using the smallest circumference between the lower ribs and iliac crests. In order to make the obesity-related data comparable with other ethnic groups, we applied the classification of World Health Organization guidelines, we defined normal weight as a BMI of 18.5~24 kg/m^2^, overweight as a BMI of 24~30 kg/m^2^, obesity as a BMI ≥30 kg/m^2^, and underweight as a BMI<18.5 kg/m^2^. Abdominal obesity was defined as a waist circumference with sex-specific criteria (WC ≥90cm in men and ≥80cm in women) (4, 16).

### Study outcomes and follow-up

The primary outcome of this study was all-cause mortality. The incidence of all-cause mortality for each participant was evaluated between June 30, 2017, and June 30, 2018. Survival time was defined as the time elapsed from initial study enrollment until the occurrence of an outcome event, kidney transplantation, and the end of the follow-up period. We obtained the survival status of the patients through periodic medical chart monitoring and contacting each patient’s dialysis unit.

### Statistical analyses

Variables are presented as mean with SD or median with interquartile range (IQR) or number (proportion) where appropriate. Baseline characteristics were compared by using the Chi-square test for categorical variables and t or Wilcoxon rank sum tests, as appropriate, for continuous variables. Restricted cubic splines were used to evaluate for nonlinear relationships between baseline BMI or WC measures and outcomes. Kaplan–Meier curves for all-cause mortality based on BMI and WC were constructed, and the log-rank test was used to compare the inter-group differences. Multivariable Cox proportional hazards regression analyses were conducted to quantify the risk of all-cause death associated with baseline BMI or WC.

We also performed a sensitivity analysis to validate the stability of our study findings. Because the patients in each BMI group may differ by WC, we further stratified the participants of each BMI group into abdominal obesity and non-abdominal obesity groups, and generated the analysis of the joint associations of BMI and WC status with all-cause mortality, with the subgroup having normal BMI and non-abdominal obesity considered as reference, adjusting for baseline covariates.

Statistical significance was set at a value of p<0.05. All analyses were performed with SPSS version 21.0 statistical software (SPSS Inc, Chicago, IL, USA) and JMP Pro 13.2 statistical software (SAS Institute Inc.)

## Results

### Basic characteristics of the participants

**Figure 1:**
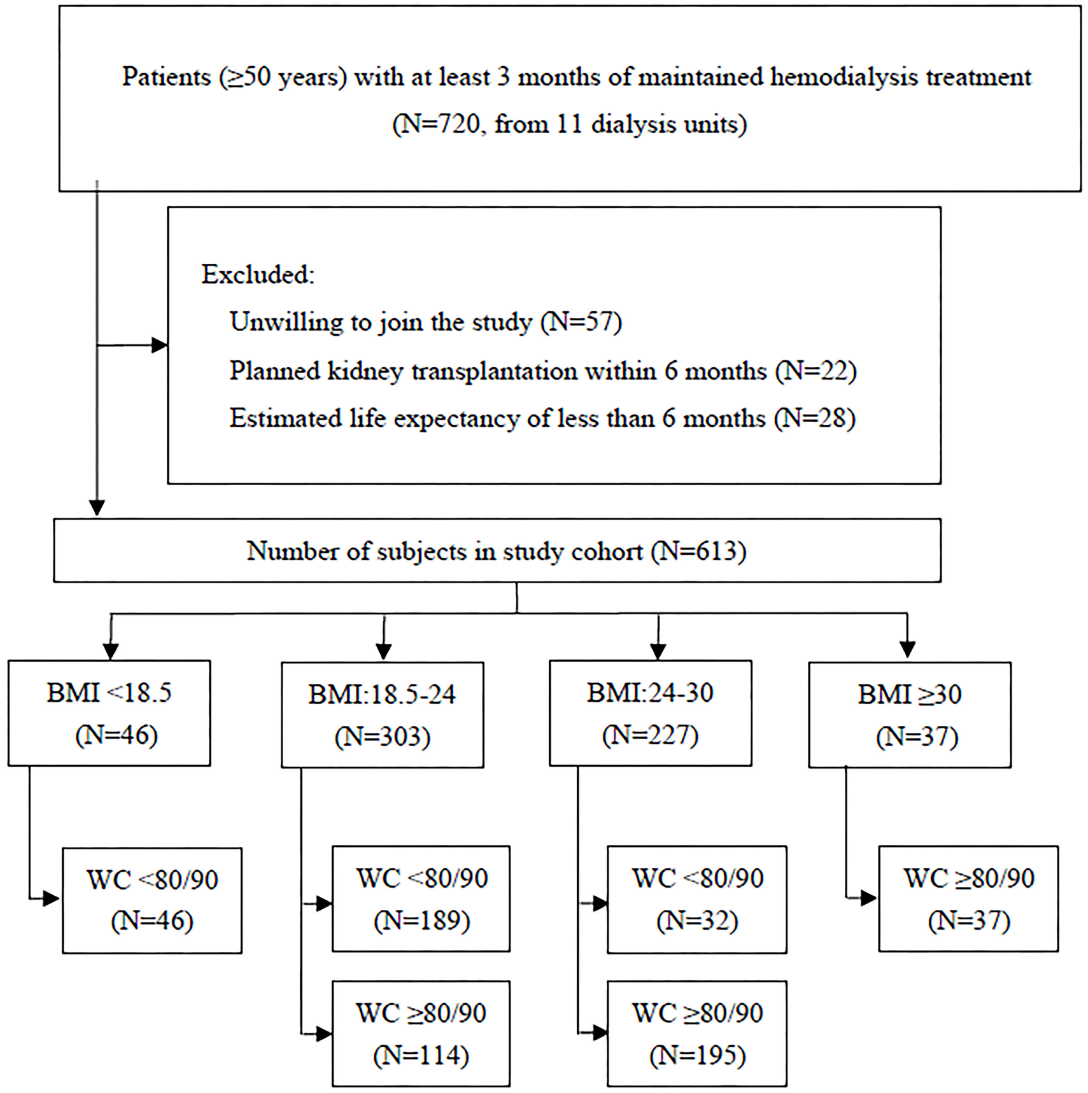
Flowchat of cohort creation.

We finally included 613 patients in this cohort study (**Figure 1**). The mean age was 63.82 ± 7.14 years, and 42.1% were women. The median hemodialysis vintage was 57 months, and the average dialysis treatment session length was 3.81 ± 0.27 h. each time, three times a week. The baseline characteristics of participants are displayed in **Table 1**. The male patients tended to have lower proportions of CHD history, abdominal obesity, shorter hemodialysis vintage, and higher level of serum creatinine and blood urea nitroge. (*p*<0.05).

**Table 1:** Baseline characteristics of the participants.

Characteristic	Total (N = 613)	Males (N=355)	Females (N=258)	*p*-value
Demographics				
Age, years	63.82 ± 7.14	63.86 ± 7.51	63.37 ± 8.14	0.444
Smoking history, n (%)	270(44.0)	150(42.3)	120(46.5)	0.294
Alcohol intake, n (%)	261(42.6)	147(41.4)	114(44.2)	0.492
Comorbidity				
Hypertension, n (%)	545(88.9)	311(87.6)	234(90.7)	0.229
Diabetes, n (%)	231(37.7)	131(36.9)	100(38.8)	0.639
Stroke, n (%)	101(16.5)	55(15.5)	46(17.8)	0.441
CHD, n (%)	194(31.6)	95(26.8)	99(38.4)	0.002
Dialysis vintage, month	57 (24–101)	54.00(21.00-91.00)	63.00 (30.00-110.50)	0.022
Single-pool Kt/V	1.29 ± 0.18	1.30 ± 0.18	1.27 ± 0.17	0.108
BMI (range), kg/m^2^	23.03(21.00-26.10)	23.00 (21.26-25.95)	23.27 (20.75-26.57)	0.638
BMI (status), n (%)				0.461
< 18.5 kg/m^2^	46(7.5)	29(8.2)	17(6.6)	
18.5–24 kg/m^2^	303(49.4)	180(50.7)	123(47.7)	
24–30 kg/m^2^	227(37.0)	124(34.9)	103(39.9)	
≥ 30 kg/m^2^	37(6.0)	22(6.2)	15(5.8)	
WC (range), cm	87.00(78.00-94.00)	86.00 (78.00-94.00)	87.00 (78.00-96.00)	0.320
Abdominal obesity, n (%)	346(56.4)	188(53.0)	158(61.2)	0.041
Laboratory data				
Hb, g/L	111.16 ± 14.53	111.09 ± 14.86	111.24 ± 14.08	0.896
Alb, g/L	39.73 ± 3.25	39.61 ± 3.18	39.90 ± 3.35	0.286
TC, mmol/L	4.25 ± 1.28	4.21 ± 0.94	4.30 ± 1.63	0.380
TG, mmol/L	2.09 ± 1.46	2.03 ± 1.30	2.19 ± 1.64	0.178
Scr, ummol/L	832.50(620.30-1006.70)	906.80(682.20-1075.70)	733.00(533.00-898.10)	<0.001
BUN, mmol/L	22.32(17.99-26.68)	23.35(19.60-27.83)	21.02(15.49-25.44)	<0.001
Calcium, mmol/L	2.24 ± 0.24	2.25 ± 0.25	2.23 ± 0.24	0.308
Phosphate, mmol/L	1.72 ± 0.65	1.71 ± 0.60	1.72 ± 0.71	0.848
iPTH, pg/mL	187.60(103.50-358.15)	176.20(99.70-332.70)	223.45(111.47-376.13)	0.093
CRP, mg/L	2.75(1.41-6.02)	2.72(1.33-4.88)	2.82(1.50-8.05)	0.080

Data were presented as mean ± SD or median (interquartile range) for continuous variables and number (%) for categorical variables. CHD, coronary heart disease; MoCA, Montreal Cognitive Assessment; Kt/V, an indicator for evaluating dialysis adequacy; BMI, body mass index; WC, waist circumference; Hb, hemoglobin; ALB, albumin; TC, total cholesterol; TG, triglyceride; Scr, serum creatinine; BUN, blood urea nitrogen; iPTH, intact parathyroid hormone; CRP, C-reactive protein.

**Figure 2:**
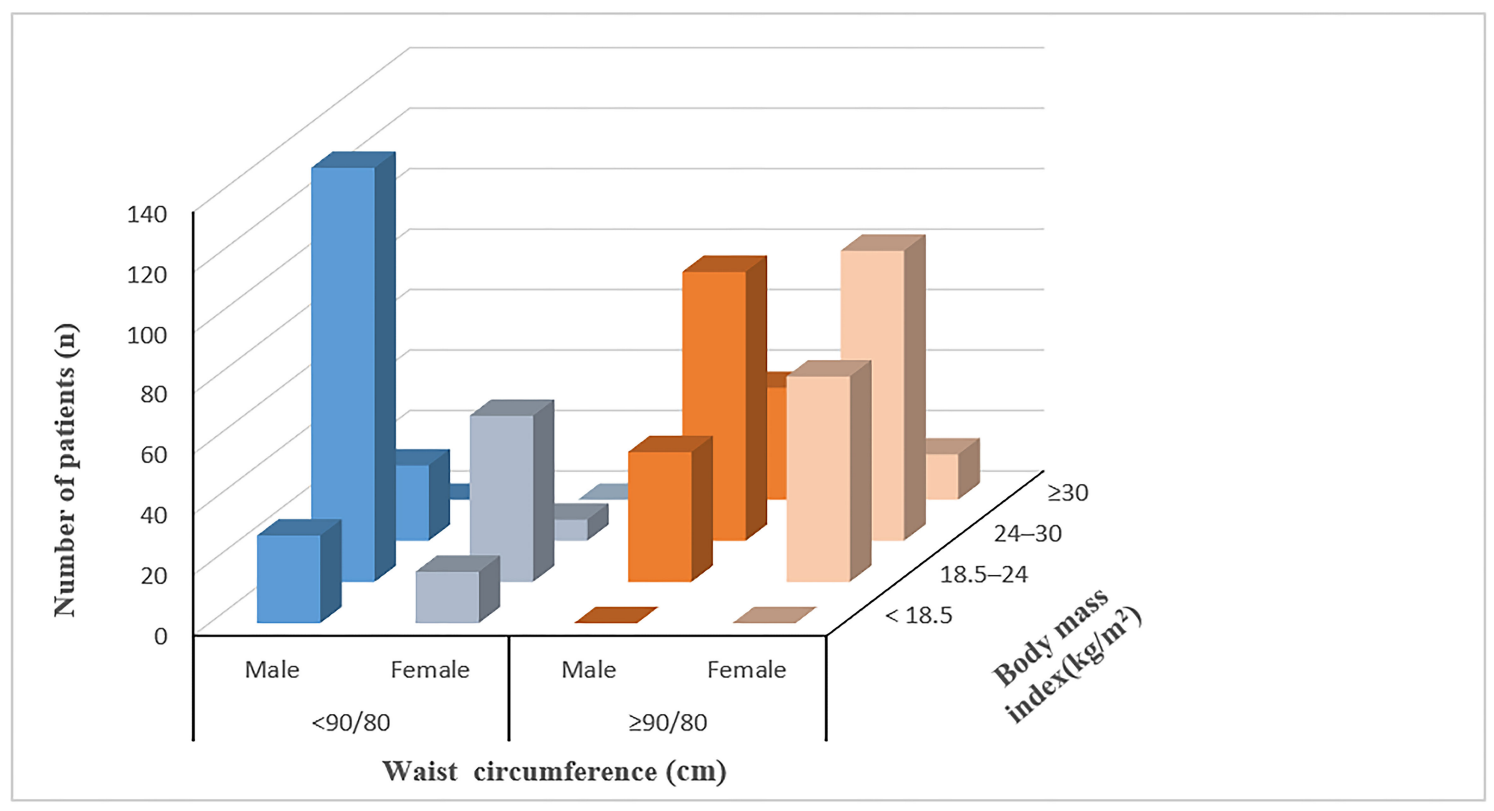
Distribution of patients according to the combination categories of BMI and WC.

Based on the baseline BMI, there were 303 (49.4%) patients with normal weight, 227 (37.0%) with overweight, 37(6.0%) with obesity, and 46 (7.5%) with underweight. Based on the baseline WC, there were 346 (56.4%) patients with abdominal obesity and 267 (43.6%) with non-abdominal obesity. The number of patients with abdominal obesity was 0 (0%) in the underweight group, 114 (37.6%) in the normal weight group, 195 (85.9%) in the overweight group, and 37 (100.0%) in the obesity group, respectively. None of the underweight patients had abdominal obesity, and all the patients in the obesity group were abdominal obesity patients. The distribution of the obesity status of the patients is shown in **Figure 2**.

### Association of BMI and WC with the risk of all-cause mortality

**Figure 3:**
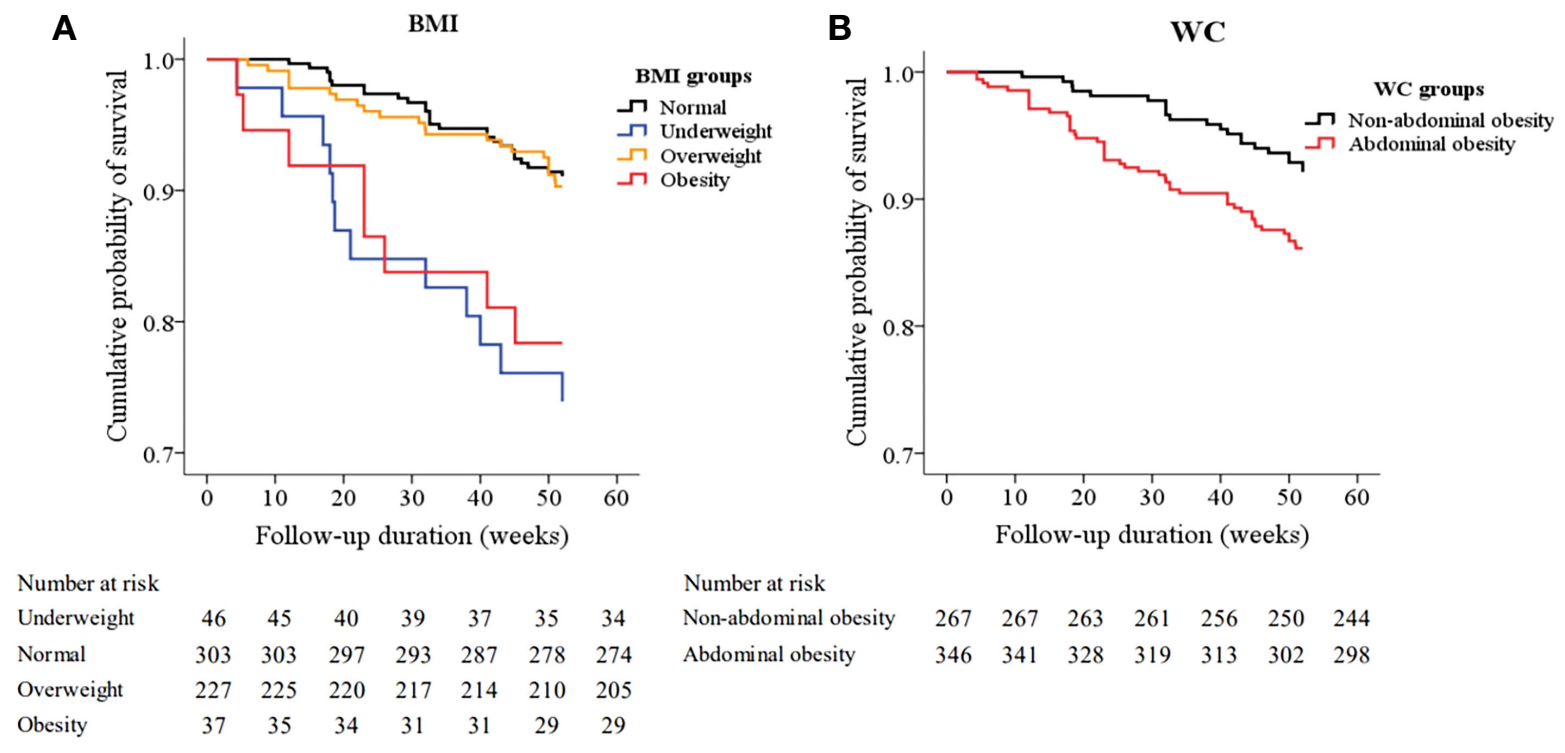
Kaplan–Meier survival curves for all-cause mortality based on body mass index (BMI) **(A)** and waist circumference (WC) **(B)**. The pairwise comparison showed that there were significant differences between the underweight group (log-rank χ^2 ^= 13.571, P<0.001) and the obesity group (log-rank χ^2 ^= 6.664 P=0.010) and the normal BMI group respectively, but there is no significant difference between the overweight group (log-rank χ^2 ^= 0.102 P=0.750) and the normal BMI group. (**Figure 3A**), and there was a significant difference between the abdominal obesity group and the non-abdominal obesity group (log-rank χ^2 ^= 5.698, P=0.017). (**Figure 3B**).

After a median follow-up of 52 weeks, all-cause death occurred in 69 patients. (). Kaplan-Meier plots showed the association between BMI categories (log-rank χ^2 ^= 18.574, *p*<0.001) and WC categories (log-rank χ^2 ^= 5.698, *p*=0.017) and all-cause death, respectively. (**Figures 3A, B**)

**Figure 4:**
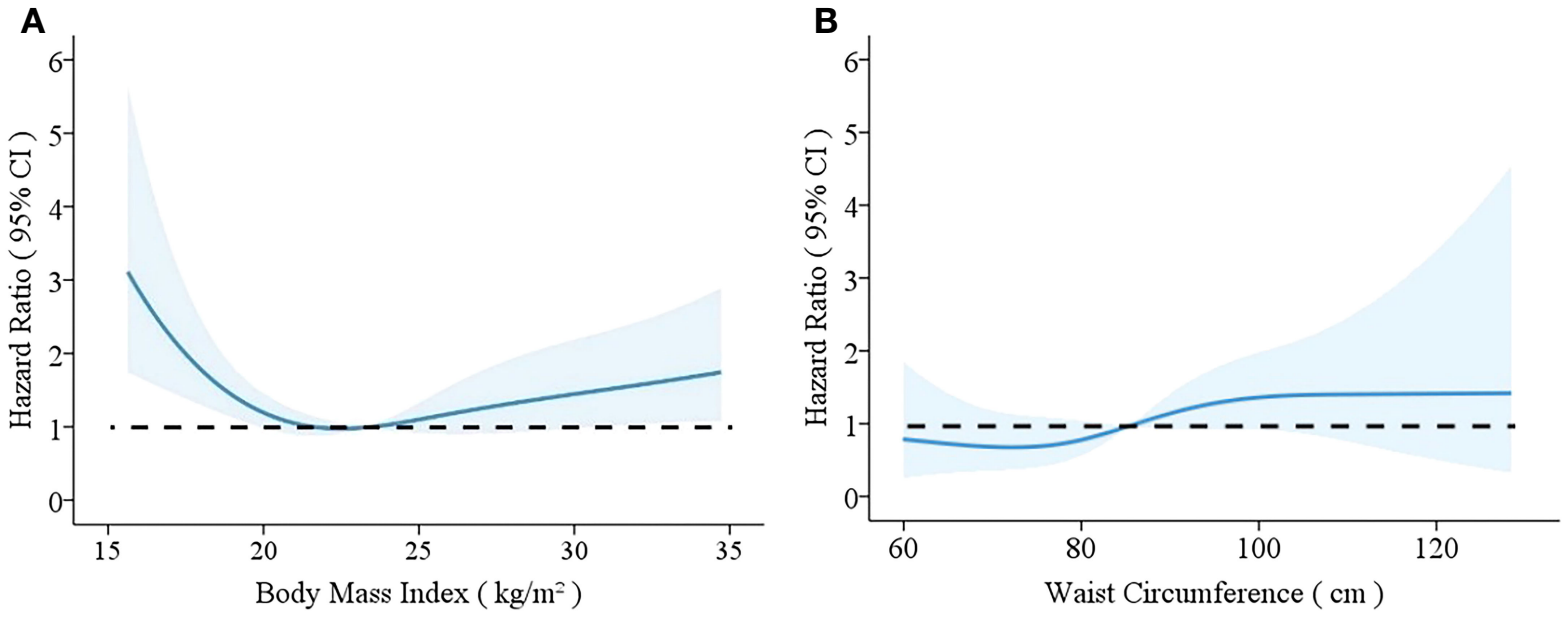
Relationships between body mass index (BMI) **(A)** and waist circumference (WC) **(B)** and mortality rates with 95% confidence intervals for hemodialysis patients. Hazard ratios for mortality depending on BMI **(A)** and WC **(B)** were modeled by separate restricted cubic splines Cox regression model analyses. The models were adjusted for age, sex, smoking, alcohol drinking, medical history of hypertension, diabetes, stroke, and CHD, dialysis vintage, Kt/V, hemoglobin, serum levels of albumin, total cholesterol, triglycerides, calcium, phosphate, intact parathyroid hormone, and C-reactive protein.

Restricted cubic splines showed the nonlinear relationships between BMI and WC and the hazard ratios for all-cause mortality. (**Figures 4A, B**). **Figure 4A** showed a U-shaped relationship between BMI and mortality, with the lowest BMI integer value of 25 kg/m^2^. Both the increase and decrease in BMI were associated with an increasing trend of death risk. **Figure 4B** shows the relationship between WC and mortality, with a hazard ratio <1.0 for WC<85cm and a hazard ratio >1.0 for WC>85cm.

Results of univariable and multivariable Cox proportional hazard analyses were summarized in **Table 2**. Using the normal weight group as a reference, multivariate Cox regression analysis showed that the risk of all-cause death tended to increase in the obesity group (HR 2.50, 95% CI, 1.12-5.59, *p*=0.026) and the underweight group (HR 3.49, 95% CI, 1.75-6.99, *p*<0.001), but not in the overweight group (HR 1.10, 95% CI, 0.62-1.93, *p*=0.758); and using the non-abdominal obesity group as a reference, the risk of all-cause death was increased in the abdominal obesity group (HR 1.80, 95% CI, 1.06-3.05, *p*=0.029).

**Table 2:** The association between BMI and WC status and all-cause mortality.

Groups	Death rate n/N (%)	Unadjusted Model		Multivariate Model 1		Multivariate Model 2	
		HR (95% CI)	*p*-Value	HR (95% CI)	*p*-Value	HR (95% CI)	*p*-Value
BMI status, kg/m^2^							
< 18.5	12/46(26.1)	3.32 (1.68-6.56)	<0.001	3.56 (1.79-7.08)	<0.001	3.49 (1.75-6.99)	<0.001
18.5–24	27/303(8.9)	1.00 (reference)		1.00 (reference)		1.00 (reference)	
24–30	22/227(9.7)	1.096(0.62-1.92)	0.750	1.025(0.58-1.80)	0.932	1.01(0.62-1.93)	0.758
≥ 30	8/37(21.6)	2.70 (1.23-5.94)	0.014	2.75 (1.24-6.14)	0.013	2.50 (1.12-5.59)	0.026
WC status, cm							
< 80/90	21/267(7.9)	1.00 (reference)		1.00 (reference)		1.00 (reference)	
≥ 80/90	48/346(13.9)	1.85(1.11-3.09)	0.019	1.75(1.04-2.92)	0.034	1.80 (1.06-3.05)	0.029

HR, hazard ratio; CI, confidence interval; BMI, body mass index; WC, waist circumference.

Unadjusted and multivariable-adjusted HRs were analyzed by the Cox proportional hazards risk model with all-cause death. Multivariable-adjusted model 1 was adjusted for age, sex, smoking, alcohol drinking, and comorbidities of hypertension, diabetes, stroke, and CHD. Multivariable-adjusted model 2 was adjusted for model 1 plus dialysis vintage, Kt/V, hemoglobin, serum levels of albumin, total cholesterol, triglycerides, calcium, phosphate, intact parathyroid hormone, and C-reactive protein.

The sensitive analysis showed the association between abdominal obesity status and all-cause mortality in different ranges of BMI. We used the group of patients with normal BMI and non-abdominal obesity as a reference, multivariate Cox regression analysis results showed that obesity (HR 5.36, 95% CI, 2.09-13.76, *p*<0.001) and underweight (HR, 5.29, 95% CI, 2.32-12.07, *p*<0.001) were still significantly associated with higher risks of all-cause mortality. In addition, for normal weight and overweight patients, the risks of all-cause mortality were also significantly increased in patients combined with abdominal obesity (HR 2.61, 95% CI, 1.20-5.66, *p*=0.016 and HR 1.79, 95% CI, 1.03-3.73, *p*=0.031, respectively), but not in patients with non-abdominal obesity (HR 0.89, 95% CI, 0.11-6.93, *p*=0.907). (**Table 3**).

**Table 3:** Associations of abdominal obesity and all-cause mortality in different ranges of BMI.

Group		Death rate n/N (%)	Unadjusted Model		Multivariate Model 1		Multivariate Model 2	
BMI, kg/m^2^	WC, cm		HR (95% CI)	*p*-Value	HR (95% CI)	*p*-Value	HR (95% CI)	*p*-Value
< 18.5	<80/90	12/46(26.1)	5.28(2.33-11.96)	<0.001	5.49(2.41-12.49)	<0.001	5.29(2.32-12.07)	<0.001
	≥80/90	–	–	–	–	–	–	–
18.5–24	<80/90	11/189(5.8)	1.00(reference)		1.00(reference)		1.00(reference)	
	≥80/90	16/114(14.0)	2.67(1.28-5.74)	0.012	2.53(1.17-5.47)	0.018	2.61(1.20-5.66)	0.016
24–30	<80/90	1/32(3.1)	0.54(0.07-4.16)	0.551	0.66(0.09-5.13)	0.692	0.89(0.11-6.93)	0.907
	≥80/90	21/195(10.8)	1.95(1.01-4.04)	0.023	1.72(1.02-4.05)	0.036	1.79(1.03-3.73)	0.031
≥30	<80/90	–	–	–	–	–	–	–
	≥80/90	8/37(21.6)	4.29(1.72-10.66)	0.002	4.36(1.73-11.02)	0.002	5.36(2.09-13.76)	<0.001

HR, hazard ratio; CI, confidence interval; BMI, body mass index; WC, waist circumference.

Unadjusted and multivariable-adjusted HRs were analyzed by the Cox proportional hazards risk model with all-cause death. Multivariable-adjusted model 1 was adjusted for age, sex, smoking, alcohol drinking, and comorbidities of hypertension, diabetes, stroke, and CHD. Multivariable-adjusted model 2 was adjusted for model 1 plus dialysis vintage, Kt/V, hemoglobin, serum levels of albumin, total cholesterol, triglycerides, calcium, phosphate, intact parathyroid hormone, and C-reactive protein.

## Discussion

In this cohort study of hemodialysis patients, we found that both underweight and obese patients defined by BMI had a significantly increased all-cause mortality compared with the patients with normal weight, while the overweight patients did not exhibit such kind of association with death. In addition, our data showed that participants with abdominal obesity defined by WC also had an increased risk of all-cause mortality compared with non-abdominal obesity counterparts even after adjusted with some potential mediators, notably, this association was observed in the patients with a wide range of BMI, even in the patients with BMI defined normal or overweight. Our data delineated the characteristics of obesity in Chinese older hemodialysis patients and provided evidence about the association between abdominal obesity and all-cause mortality in different BMI ranges.

Currently, obesity has been regarded as a kind of chronic disease, and obesity-related issues in patients with chronic kidney disease also cause great attention (9, 10). In 2002, the prevalence of obesity in the U.S. Renal Data System was 29% (17). Data from some European countries showed that the proportion of obese patients on dialysis was less than in the U.S., ranging from 10% to 12% (18). In China, the latest national survey showed that the prevalence was 34.3% for overweight and 16.4% for obesity in the general population from 2015 to 2019, and the study also found that there is a trend that Chinese people with obesity tend to have abdominal obesity (apple-shaped) rather than gluteofemoral obesity (pear-shaped) (19). In our study, the proportion of overweight was 37% and obesity was 6% defined by BMI; we also noticed that the total abdominal obesity had reached 56.4%. Notably, all of the obese patients defined by increased BMI in our study also had abdominal obesity, and abdominal obesity even existed in the normal weight and overweight groups defined by BMI, that is to say, the main feature of obesity in this group of elder patients undergoing hemodialysis was abdominal obesity, these results also mirrored the differences between the general population and patients undergoing hemodialysis in China. Although most of the current understanding of the negative influence of excess body fat on health was built up on the basis of the data on general obesity, which was defined by increased BMI (13, 16, 20), evidence is emerging that BMI is an imperfect metric for obesity, and WC appears to be a better indicator of this disease than BMI (21–23). A pooled analysis included 650000 white adults from the Mayo Clinic indicated that abdominal obesity defined by increased WC was positively associated with higher all-cause mortality at all levels of BMI from 20 to 50 kg/m^2^ (24). These data provide evidence that abdominal obesity is an independent risk factor for all-cause mortality in community individuals, based on such related information, we used WC to evaluate the status of abdominal obesity on the basis of BMI classification, and this is also the first report about the features of obesity among Chinese elder hemodialysis patients.

Another major concern is the relationship between obesity and adverse outcomes in hemodialysis patients. Maleeka (5) made a meta-analysis that included 852162 patients undergoing hemodialysis in 65 cohorts, the results showed that a 1 kg/m^2^ increase in BMI was associated with a 3 and 4% reduction in all-cause and cardiovascular mortality in patients on hemodialysis, the results seemed to indicate that being obese is protective against all-cause and cardiovascular mortality. However, some studies didn’t show this “obesity paradox”. Ellen (25) found a U-shaped association between obesity measured by BMI and all-cause mortality after 7 years of follow-up in a group of hemodialysis patients who were younger than 65 years old. Like the limitation in the general population, using BMI in the evaluation of obesity in dialysis patients also couldn’t reflect the real situation of abdominal fat deposition (23). By applying both BMI and WC measurements, our results showed that abdominal obesity existed in individuals with a wide range of BMI, even in the patients with BMI defined as normal or overweight, this result was in accordance with the results from the cohort study from Korean, they included the data of 18,699 adult hemodialysis patients who were followed up for 4 years and for whom BMI and WC information were available, participants with the highest WC had a higher risk of mortality, these data in Asian hemodialysis population reflected the important in monitoring the WC among hemodialysis patients (26).

As abdominal obesity was the marker of visceral fat accumulation, previous studies indicated that it was more strongly associated with multiple chronic diseases than gluteofemoral obesity (27, 28). This overlapped classification of obesity between BMI and WC measurements in our study might partially explain the reason why we didn’t see the “obesity paradox” in these participants, at the same time, our data also indicated that visceral fat accumulation played an important role in deciding the relationship of obesity and all-cause mortality, it seems necessary to use BMI and WC at the same time in evaluating the obesity status in hemodialysis patients, this combining metrics of overall body size with BMI and local fat accumulation with WC seems to be particularly important in predicting the clinical outcomes in patients undergoing hemodialysis.

Our study has various strengths. We used standardized data from a prospective cohort of participants undergoing hemodialysis from 11 centers, all participants had relatively stable states with proper dry weight at the enrollment, which significantly reduced the influence of fluid overload on the evaluation of obesity. Furthermore, both BMI and WC applied in our study are simple and reliable metrics for different types of obesity, which provide an accurate classification of obesity status in our study. Nevertheless, several limitations should be recognized, including a relatively small sample size and short time of follow-up, the representative of the enrolled patients was only restricted within Beijing which may lead to selection bias, and some larger cohorts are needed to delineate the characteristics of obesity in patients undergoing hemodialysis in China. In addition, spectroscopic bioimpedance has not yet been used in this study, and it is crucial in determining the distribution of fat and muscle mass. The results of this study suggested that patients undergoing hemodialysis might improve life expectancy by proper weight control to the recommended levels, and both BMI and WC measurements are important metrics in identifying obesity status which should be used simultaneously in the obesity-related evaluation.

## Conclusions

In conclusion, our findings indicate that abdominal obesity is common and associated with all-cause mortality among middle-aged and older age Chinese patients undergoing hemodialysis. The obese patients defined by increased BMI also have abdominal obesity, which reflects that visceral fat deposition is a key feature among this group of patients, it might be necessary to make a combined measurement of BMI and WC while evaluating of obesity status in these patients.

## Data availability statement

The raw data supporting the conclusions of this article will be made available by the authors, without undue reservation.

## Ethics statement

The studies involving humans were approved by Beijing Shijitan Hospital ethics committee. The studies were conducted in accordance with the local legislation and institutional requirements. The participants provided their written informed consent to participate in this study.

## Author contributions

ZS: Writing - original draft. YG: Data curation, Writing - review & editing. PY: Data curation, Methodology, Software, Conceptualization, Writing - original draft. YL: Conceptualization, Supervision, Writing - original draft.

## References

[1] Jaacks LM Vandevijvere S Pan A McGowan CJ Wallace C Imamura F. The obesity transition: stages of the global epidemic. Lancet Diabetes Endocrinol (2019) 7:231–40. doi: 10.1016/S2213-8587(19)30026-9 PMC7360432 30704950

[2] Health TLP. Tackling obesity seriously: the time has come. Lancet Public Health (2018) 3:e153. doi: 10.1016/S2468-2667(18)30053-7 29627076

[3] Williams M Lee M Alfadhel M Kerr AJ. Obesity and all-cause mortality following acute coronary syndrome (ANZACS-QI 53). Heart Lung Circ (2021) 30:1854–62. doi: 10.1016/j.hlc.2021.04.014 34083149

[4] Flegal KM Kit BK Orpana H Graubard BI. Association of all-cause mortality with overweight and obesity using standard body mass index categories: a systematic review and meta-analysis. JAMA. (2013) 309:71–82. doi: 10.1001/jama.2012.113905 23280227 PMC4855514

[5] Ladhani M Craig JC Irving M Clayton PA Wong G. Obesity and the risk of cardiovascular and all-cause mortality in chronic kidney disease: a systematic review and meta-analysis. Nephrol Dial Transplant. (2017) 32:439–49. doi: 10.1093/ndt/gfw075 27190330

[6] Hall YN Xu P Chertow GM. Relationship of body size and mortality among US Asians and Pacific Islanders on dialysis. Ethn Dis (2011) 21:40–6. PMC4115365 21462728

[7] Postorino M Marino C Tripepi G Zoccali C. Abdominal obesity and all-cause and cardiovascular mortality in end-stage renal disease. J Am Coll Cardiol (2009) 53:1265–72. doi: 10.1016/j.jacc.2008.12.040 19358939

[8] Pujilestari CU Nyström L Norberg M Ng N. Waist circumference and all-cause mortality among older adults in rural Indonesia. Int J Environ Res Public Health (2019) 16:116. doi: 10.3390/ijerph16010116 30609857 PMC6339011

[9] Chen Y Yang Y Jiang H Liang X Wang Y Lu W. Associations of BMI and waist circumference with all-cause mortality: A 22-year cohort study. Obesity. (2019) 27:662–9. doi: 10.1002/oby.22423 30861324

[10] Seidell JC. Waist circumference and waist/hip ratio in relation to all-cause mortality, cancer and sleep apnea. Eur J Clin Nutr (2010) 64:35–41. doi: 10.1038/ejcn.2009.71 19639001

[11] Castro A Bazanelli AP Nerbass FB Cuppari L Kamimura MA. Waist circumference as a predictor of mortality in peritoneal dialysis patients: a follow-up study of 48 months. Br J Nutr (2017) 117:1299–303. doi: 10.1017/S0007114517001179 28583215

[12] Lowrie EG Li Z Ofsthun N Lazarus JM. Body size, dialysis dose and death risk relationships among hemodialysis patients. Kidney Int (2002) 62:1891–7. doi: 10.1046/j.1523-1755.2002.00642.x 12371994

[13] Kalantar-Zadeh K Kopple JD Kilpatrick RD McAllister CJ Shinaberger CS Gjertson DW. Association of morbid obesity and weight change over time with cardiovascular survival in hemodialysis population. Am J Kidney Dis (2005) 46:489–500. doi: 10.1053/j.ajkd.2005.05.020 16129211

[14] Luo Y Murray AM Guo YD Tian R Ye PP Li X. Cognitive impairment and associated risk factors in older adult hemodialysis patients: a cross-sectional survey. Sci Rep (2020) 10:12542. doi: 10.1038/s41598-020-69482-1 32719428 PMC7385128

[15] Guo Y Tian R Ye P Luo Y. Frailty in older patients undergoing hemodialysis and its association with all-cause mortality: A prospective cohort study. Clin Interv Aging. (2022) 17:265–75. doi: 10.2147/CIA.S357582 PMC8934156 35313671

[16] Safaei M Sundararajan EA Driss M Boulila W Shapi'i A. A systematic literature review on obesity: Understanding the causes & consequences of obesity and reviewing various machine learning approaches used to predict obesity. Comput Biol Med (2021) 136:104754. doi: 10.1016/j.compbiomed.2021.104754 34426171

[17] Kramer HJ Saranathan A Luke A Durazo-Arvizu RA Guichan C Hou S. Increasing body mass index and obesity in the incident ESRD population. J Am Soc Nephrol. (2006) 17:1453–9. doi: 10.1681/ASN.2005111241 16597682

[18] Bossola M Giungi S Tazza L Luciani G. Is there any survival advantage of obesity in Southern European haemodialysis patients. Nephrol Dial Transplant. (2010) 25:318–9. doi: 10.1093/ndt/gfp543 19892755

[19] Pan XF Wang L Pan A. Epidemiology and determinants of obesity in China. Lancet Diabetes Endocrinol (2021) 9:373–92. doi: 10.1016/S2213-8587(21)00045-0 34022156

[20] Bouchard CBMI. fat mass, abdominal adiposity and visceral fat: where is the 'beef'. Int J Obes (Lond). (2007) 31:1552–3. doi: 10.1038/sj.ijo.0803653 17549092

[21] Rothman KJ. BMI-related errors in the measurement of obesity. Int J Obes (Lond). (2008) 32 Suppl 3:S56–9. doi: 10.1038/ijo.2008.87 18695655

[22] Nimptsch K Konigorski S Pischon T. Diagnosis of obesity and use of obesity biomarkers in science and clinical medicine. Metabolism. (2019) 92:61–70. doi: 10.1016/j.metabol.2018.12.006 30586573

[23] Goh VH Tain CF Tong TY Mok HP Wong MT. Are BMI and other anthropometric measures appropriate as indices for obesity? A study in an Asian population. J Lipid Res (2004) 45:1892–8. doi: 10.1194/jlr.M400159-JLR200 15258201

[24] Cerhan JR Moore SC Jacobs EJ Kitahara CM Rosenberg PS Adami HO. A pooled analysis of waist circumference and mortality in 650,000 adults. Mayo Clin Proc (2014) 89:335–45. doi: 10.1016/j.mayocp.2013.11.011 PMC4104704 24582192

[25] Hoogeveen EK Halbesma N Rothman KJ Stijnen T van Dijk S Dekker FW. Obesity and mortality risk among younger dialysis patients. Clin J Am Soc Nephrol. (2012) 7:280–8. doi: 10.2215/CJN.05700611 PMC3280032 22223612

[26] Kim CS Han KD Choi HS Bae EH Ma SK Kim SW. Association of body mass index and waist circumference with all-cause mortality in hemodialysis patients. J Clin Med (2020) 9:1289. doi: 10.3390/jcm9051289 32365666 PMC7288310

[27] Ibrahim MM. Subcutaneous and visceral adipose tissue: structural and functional differences. Obes Rev (2010) 11:11–8. doi: 10.1111/j.1467-789X.2009.00623.x 19656312

[28] Karpe F Pinnick KE. Biology of upper-body and lower-body adipose tissue–link to whole-body phenotypes. Nat Rev Endocrinol (2015) 11:90–100. doi: 10.1038/nrendo.2014.185 25365922

